# Human umbilical cord mesenchymal stromal cells-derived extracellular vesicles exert potent bone protective effects by CLEC11A-mediated regulation of bone metabolism

**DOI:** 10.7150/thno.39238

**Published:** 2020-01-16

**Authors:** Yin Hu, Yan Zhang, Chu-Yu Ni, Chun-Yuan Chen, Shan-Shan Rao, Hao Yin, Jie Huang, Yi-Juan Tan, Zhen-Xing Wang, Jia Cao, Zheng-Zhao Liu, Ping-Li Xie, Ben Wu, Juan Luo, Hui Xie

**Affiliations:** 1 Department of Orthopedics, Xiangya Hospital, Central South University, Changsha, Hunan 410008, China.; 2 Movement System Injury and Repair Research Center, Xiangya Hospital, Central South University, Changsha, Hunan 410008, China.; 3 Department of Metabolism and Endocrinology, The First Affiliated Hospital, University of South China, Hengyang, Hunan 421001, China.; 4 Department of Sports Medicine, Xiangya Hospital, Central South University, Changsha, Hunan 410008, China.; 5 Xiangya Nursing School, Central South University, Changsha, Hunan 410013, China.; 6 Department of Forensic Science, School of Basic Medical Science, Central South University, Changsha, Hunan 410013, China.; 7 Hunan Key Laboratory of Organ Injury, Aging and Regenerative Medicine, Changsha, Hunan 410008, China.; 8 Hunan Key Laboratory of Bone Joint Degeneration and Injury, Changsha, Hunan 410008, China.; 9 National Clinical Research Center for Geriatric Disorders, Xiangya Hospital, Central South University, Changsha, Hunan 410008, China.

**Keywords:** extracellular vesicles, umbilical cord-derived mesenchymal stromal cells, bone homeostasis, osteoporosis, CLEC11A

## Abstract

Osteoporosis and osteoporotic fractures severely compromise quality of life in elderly people and lead to early death. Human umbilical cord mesenchymal stromal cell (MSC)-derived extracellular vesicles (hucMSC-EVs) possess considerable therapeutic effects in tissue repair and regeneration. Thus, in the present study, we investigated the effects of hucMSC-EVs on primary and secondary osteoporosis and explored the underlying mechanisms.

**Methods**: hucMSCs were isolated and cultured. EVs were obtained from the conditioned medium of hucMSCs and determined by using transmission electron microscopy, dynamic light scattering and Western Blot analyses. The effects of hucMSC-EVs on ovariectomy-induced postmenopausal osteoporosis and tail suspension-induced hindlimb disuse osteoporosis in mouse models were assessed by using microcomputed tomography, biomechanical, histochemical and immunohistochemical, as well as histomorphometric analyses. Proteomic analysis was applied between hucMSC-EVs and hucMSCs to screen the candidate proteins that mediate hucMSC-EVs function. The effects of hucMSC-EVs on osteogenic and adipogenic differentiation of bone marrow mesenchymal stromal cells (BMSCs), and osteoclastogenesis of the macrophage cell line RAW264.7 *in vitro* were determined by using cytochemical staining and quantitative real-time PCR analysis. Subsequently, the roles of the key protein in hucMSC-EVs-induced regulation on BMSCs and RAW264.7 cells were evaluated.

**Results:** hucMSCs were able to differentiate into osteoblasts, adipocytes or chondrocytes and positively expressed CD29, CD44, CD73 and CD90, but negatively expressed CD34 and CD45. The morphological assessment revealed the typical cup- or sphere-shaped morphology of hucMSC-EVs with diameters predominantly ranging from 60 nm to 150 nm and expressed CD9, CD63, CD81 and TSG101. The systemic administration of hucMSC-EVs prevented bone loss and maintained bone strength in osteoporotic mice by enhancing bone formation, reducing marrow fat accumulation and decreasing bone resorption. Proteomic analysis showed that the potently pro-osteogenic protein, CLEC11A (C-type lectin domain family 11, member A) was very highly enriched in hucMSC-EVs. In addition, hucMSC-EVs enhanced the shift from adipogenic to osteogenic differentiation of BMSCs via delivering CLEC11A *in vitro*. Moreover, CLEC11A was required for the inhibitory effects of hucMSC-EVs on osteoclast formation.

**Conclusion:** Our results suggest that hucMSC-EVs serve as a critical regulator of bone metabolism by transferring CLEC11A and may represent a potential agent for prevention and treatment of osteoporosis.

## Introduction

Bone, a dynamic vital organ, undergoes modeling and remodeling throughout life that constantly responds to physiological and pathological stress and stimulation 1,2. During bone remodeling, old or damaged bone is digested by osteoclasts (sole bone-resorbing cells) at a specific site where growth factors are released from the bone matrix to induce recruitment of bone marrow mesenchymal stromal cells (BMSCs) 1,2. Subsequently, BMSCs differentiate into osteoblasts and new bone is formed 1,2. In normal conditions, osteoclastic bone resorption is coupled with osteoblastic bone formation 2. Likewise, bone formation is tightly linked with adipogenesis in the bone marrow microenvironment due to the fact that osteoblasts and adipocytes share the common progenitors, BMSCs 3. Osteogenic and adipogenic differentiation of BMSCs require a proper balance, which is regulated and maintained by intracellular and extracellular signals 4. In pathological conditions, including estrogen deficiency, aging, disuse, drug use and malnutrition, bone formation and resorption are uncoupled or/and osteoblast differentiation and adipocyte differentiation of BMSCs are imbalanced, which often results in metabolic bone diseases such as osteoporosis 5,6. Therefore, therapeutic strategies designed to orchestrate osteoclastic-blastic coupling or osteo-adipogenic balance will be promising for preventing and treating osteoporosis.

Stem cell therapy has been demonstrated as an effective approach for bone tissue repair and regeneration including osteoporosis 7-9. Human umbilical cord-derived mesenchymal stromal cells (hucMSCs) are considered to be significantly superior to stem cells from other sources, since they have a more readily available source, non-invasive collection procedure, powerful immunomodulation capacity and prominent self-renewal property 10,11. In recent years, accumulating evidences have proved that transplanted stem cells exert the therapeutic effects by paracrine mechanisms to regulate the function properties of tissue-resident recipient cells 11,12. Orthotopic transplantation of hucMSCs has been found to remarkably increase new bone formation in calvarial defects by activating host cells and endogenous repair mechanisms 13. The culture supernatant of hucMSCs has been verified to promote bone regeneration of femoral shaft defects through modulating the expression of bone cells related cytokines 14. These studies suggest that the pro-osteogenesis ability of hucMSCs may be mediated via secreted beneficial factors.

Extracellular vesicles (EVs), released by various cells types, are membrane-enclosed vesicles that can be broadly categorized into 3 main classes: exosomes, microvesicles and apoptotic bodies 15-18. Exosomes and microvesicles are the most remarkably described classes of EVs, which mediate cell paracrine actions by transferring bioactive molecules, including nucleic acids, proteins and lipids, into target cells to modulate their activities 19-21. It has been reported that EVs are able to exhibit the therapeutic efficacy similar to their parent cells 22-24. Considering the positive effects of hucMSCs on bone regeneration, we hypothesized that EVs derived from hucMSCs (hucMSC-EVs) might also promote bone formation, thus preventing or treating osteoporosis. Studies have shown that hucMSC-EVs are capable of alleviating tissue injuries and promoting regenerative responses in variety of diseases models 11,23. Zhang *et al*. recently also found that local transplantation of hucMSC-EVs could accelerate fracture healing through hypoxia inducible factor-1α-mediated promotion of angiogenesis 25. However, to date, no research has explored the therapeutic potential of hucMSC-EVs in osteoporosis.

In this study, we investigated the effects of hucMSC-EVs on bone mass and strength in mouse osteoporotic models and evaluated the impacts of hucMSC-EVs on bone metabolism *in vivo* and *in vitro*. Moreover, we used proteomic analysis to screen the key protein that may mediate hucMSC-EVs function, and preliminarily clarified its roles in hucMSC-EVs-induced multiple regulation of bone metabolism.

## Materials and Methods

### Isolation, culture and identification of hucMSCs

This study was approved by the Ethical Review Board at Xiangya Hospital of Central South University, and informed consent was obtained from all participants before sample collection. Human umbilical cord samples (n = 3) were obtained from healthy mothers after childbirth and processed within 6 h of collection. hucMSCs were isolated from fresh umbilical cord tissues as previously described 26. Briefly, umbilical cords were firstly washed with phosphate buffer saline (PBS) containing penicillin and streptomycin for twice to remove the blood. The rinsed cords were cut into 3-4 cm long segments in culture dishes and cord vessels were pulled away. Then, the cord segments were minced into pieces and floated in a DMEM/F-12 (Gibco, Grand Island, USA) supplemented with 10% fetal bovine serum (FBS; Gibco), 1% GlutaMAX (Gibco), 100 U/mL penicillin and 100 μg/mL streptomycin (Gibco). The cord pieces were incubated at 37°C with 5% CO_2_ in a humidified environment and left undisturbed for 72 h, after which fresh complete medium was added. Subsequently, half of the medium was replaced every 3 days and the cord tissue was removed after colonies of fibroblast-like cells appeared. Cells were trypsinized and passaged until they reached 80-90% confluence. Early-passage hucMSCs (passages 2-6) were used for the follow-up experiments.

The morphology of hucMSCs was observed by an inverted microscope (Leica DMI6000B, Solms, Germany). The osteogenic, adipogenic and chondrogenic differentiation potentials of hucMSCs were determined by using the respective differentiation medium (Cyagen Biosciences Inc, Guangzhou, China) as described previously 27,28. Osteogenesis of hucMSCs was examined using Alizarin Red S (ARS) staining to test calcium depositions at day 14 of induction, adipogenesis of hucMSCs was evaluated using Oil Red O (ORO) staining to observe lipid droplets at day 21 of induction, and chondrocyte differentiation of hucMSCs was assessed by Alcian Blue staining of extracellular matrix at day 28 of induction. Immunophenotype of hucMSCs was analysed by flow cytometry with the fluorescein- conjugated monoclonal antibodies (CD29, CD34, CD44, CD45, CD73 and CD90) according the manufacturer's protocol. Fluorescein-labeled isotype-matched antibodies were used as the negative controls. All antibodies were purchased from BD Biosciences (San Jose, USA).

### Isolation and characterization of hucMSC-EVs

hucMSC-EVs were purified as previously described 29. Briefly, hucMSCs were cultured in fresh complete medium containing EVs-free FBS for 48 h. The conditioned medium of hucMSCs was collected and sequentially centrifuged at 300 ×g for 10 min, 2000 ×g for 30 min and 10,000 ×g for 30 min to eliminate dead cells and cellular debris. Then, the supernatant was filtered through a 0.22 μm filter (Millipore, Billerica, USA). 12 mL supernatant was concentrated to approximately 1 mL by ultra-filtration with an Amicon Ultra-15 Centrifugal Filter Unit (100 kDa; Millipore) at 4000 ×g. The ultrafiltration supernatant was washed with PBS for twice and re-ultrafiltrated to 1 mL. Exoquick-TC Exosome Precipitation Solution (System Biosciences, Palo Alto, USA) was mixed with the ultrafiltration liquid in proportion. After incubation overnight, the mixture was centrifuged at 1500 ×g to completely remove supernatant. hucMSC-EVs pellets were resuspended in a right amount of PBS. All procedures were performed at 4°C. Protein content of hucMSC-EVs was assayed by a BCA Protein Assay Kit (Thermo Fisher Scientific, Waltham, USA). EVs were stored at -80°C for later use.

The morphology of hucMSC-EVs was observed by transmission electron microscope (TEM) (Hitachi, Tokyo, Japan). The EV size distribution was determined using a Nanosizer™ instrument (Malvern Instruments, Malvern, UK). The expression of exosomal surface markers (CD9, CD63, CD81 and TSG101) in hucMSC-EVs was identified by Western Blot. hucMSCs were used as the control.

### Osteoporosis models and treatments

All animal procedures were approved by the Ethical Review Board at Xiangya Hospital of Central South University. For postmenopausal osteoporosis model, 8-week-old female C57BL/6 mice were randomly divided into 3 groups (n = 10 per group) and subjected to either bilateral ovariectomy (OVX) or a sham operation (Sham). hucMSC-EVs (100 μg dissolved in 100 μl PBS) or vehicle (100 μl PBS) were intravenously injected via the tail vein once a week starting one week after the surgery. Two months later, the mice were anesthetized and sacrificed after blood collection by enucleation of eyeball. The blood was centrifuged at 1,000 ×g for 15 min to harvest serum samples, which was stored at -80°C for later use. Uteri were dissected and weighed to verify the success of OVX operation. The femurs were collected for further experiments. For senile osteoporosis model, 16-month-old male C57BL/6 mice were randomly divided into 2 groups (n = 8 per group) and injected intravenously with hucMSC-EVs (100 μg dissolved in 100 μl PBS) or vehicle (100 μl PBS) through the tail vein once a week for 3 months. All mice were sacrificed and the femur specimens were obtained for further analysis. For disuse osteoporosis model, 3-month-old C57BL/6 mice were randomly divided into 3 groups (n = 10 per group) and suffered from hindlimb-unloading induced by tail suspension (TS) or hindlimb-loading (control group). hucMSC- EVs (100 μg dissolved in 100 μl PBS) or vehicle (100 μl PBS) were intravenously administrated via the tail vein twice a week for 21 days. The mice were sacrificed after treatment, and the blood and femur samples were collected for the downstream measurements.

### Microcomputed tomography (μCT) analysis

The dissected femurs were fixed in 4% paraformaldehyde (PFA) for 48 h and stored in PBS at 4°C. High-resolution μCT (Skyscan 1176; Skycan, Aartselaar, Belgium) was used to scan femurs at a resolution of 8.88 μm per pixel, with voltage of 50 kV and current of 400 μA. The distal femurs were used to analyze trabecular bone parameters. The region of interest (ROI) was selected starting from 0.45 mm below the distal growth plate and extended for 0.45 mm proximally to measure the trabecular bone volume fraction (Tb. BV/TV), trabecular thickness (Tb. Th) and trabecular number (Tb. N). The mid-diaphysis femurs were used to analyze cortical bone parameters. 5% of femoral length was selected as ROI to measure the cortical thickness (Ct. Th).

### Biomechanical analysis

To evaluate biomechanical properties of the femurs, three-point bending test was performed using a computer-controlled mechanical testing machine (WD-D1; Shanghai Zhuoji Instruments Co.Ltd., Shanghai, China). The femora were placed on the lower two supporting rods (each support point was 4 mm from the midpoint of the samples) and loaded to fracture under displacement control (5 mm/min). The maximum load (N) was calculated and recorded according to the load-deformation curves.

### Histochemistry, immunohistochemistry and histomorphometry analyses

Histochemical and immunohistochemical stainings were performed as previously described 30,31. Briefly, dissected bones were fixed in 4% PFA for 48 h, decalcified in 0.5 M EDTA (pH = 7.4) for 4-5 days, and dehydrated in graded ethanol, before being embedded in paraffin. 5-μm-thick bone sections were stained with hematoxylin and eosin (H&E), osteocalcin (OCN) and tartrate-resistant acid phosphatase (TRAP) to quantify number and area of adipocytes, number of osteoblasts, and number of osteoclasts, respectively. Images were photographed using an Olympus CX31 microscope (Olympus, Hamburg, Germany). Primary antibody against OCN and the secondary antibody were purchased from Abcam (Cambridge, Britain). TRAP Staining Kit was obtained from Sigma (St. Louis, USA).

Histomorphometric analysis was conducted to test dynamic bone formation. Briefly, the mice were intraperitoneally injected with 10 mg/kg body weight calcein (Sigma) dissolved in PBS at 10 and 3 days before sacrifice. The collected femurs were fixed in 4% PFA for 48 h, dehydrated in increasing concentrations of ethanol and sectioned without decalcification (60-μm sections). Calcein double labeling was observed by a fluorescence microscope (Leica). Mineral apposition rates (MAR) of trabecular bone were determined using Image-Pro Plus 6 software.

### Enzyme-linked immunosorbent assay (ELISA)

The rate of bone formation and bone resorption was evaluated by measuring serum levels of OCN and C-terminal telopeptides of type I collagen (CTX-I) proteins respectively, using commercial ELISA kits (Elabscience, Wuhan, China). The optical density was detected with a microplate reader (Bio-Rad 680, Hercules, USA). All the procedures were performed according to the manufacturer's instructions.

### Proteomic analysis

#### Samples preparation and protein digestion

Protein samples were prepared as described previously 29. Briefly, hucMSCs in three biological replicates (called C1, C2 and C3) were seeded in culture flasks and incubated in fresh EVs-free complete medium for 48 h after reaching 80% confluence. hucMSCs and their respective conditioned medium were collected. EVs samples (E1, E2 and E3) were harvested from the culture medium of corresponding hucMSCs. The cells and EVs samples were processed for label-free quantitative proteomic analysis by Jingjie PTM BioLab (Hangzhou, China). Lysis buffer (8 M urea, 1% Protease Inhibitor Cocktail) was added to the samples, followed by sonication three times on ice. The remaining debris was removed by centrifugation at 12,000 g for 10 min at 4°C. Then, the supernatant was collected and the protein concentration was detected with a BCA kit. For trypsin digestion, the protein samples were reduced with 5 mM dithiothreitol at 56°C for 30 min and alkylated with 11 mM iodoacetamide at room temperature for 15 min in darkness. 100 mM NH4HCO3 was added to the protein solution to dilute urea concentration (less than 2M). Subsequently, trypsin was added into the samples at 1:50 trypsin-to-protein mass ratio for the first digestion overnight and 1:100 trypsin-to-protein mass ratio for a second 4 h-digestion.

#### HPLC fractionation

High pH reverse-phase HPLC was used to fractionate the tryptic peptides with Agilent 300 Extend C18 column (5 μm particles, 4.6 mm ID, 250 mm length). Peptides were separated into 60 fractions with a gradient of 8% to 32% acetonitrile (pH 9.0) over 60 min, and then combined into 4 fractions and dried by vacuum centrifuging.

#### Quantitative proteomic analysis by LC-MS/MS

The LC-MS/MS analysis was performed on an EASY-nLC Liquid Chromatograph (Thermo) coupled with a Q Exactive mass spectrometer (Thermo) as previously described 29,32. Briefly, Peptides were dissolved in 0.1% formic acid (solvent A) and separated on a home-made reversed-phase analytical column (15 cm length, 75 μm i.d.). The gradient was comprised of an increase from 8% to 24% solvent B (0.1% formic acid in 90% acetonitrile) over 40 min, 24% to 36% in 14 min and climbing to 80% in 3 min then holding at 80% for the last 3 min, all at a flow rate of 400 nl/min on an EASY-nLC 1000 UPLC system. Subsequently, the peptides were ionized using NSI source followed by tandem mass spectrometry (MS/MS) in Q Exactive^TM^ Plus (Thermo). The voltage of electrospray applied was 2.0 kV. The m/z scan range was 350 to 1800 for full scan, and intact peptides were detected in the Orbitrap at a resolution of 70,000. Peptides were then selected for MS/MS using NCE setting as 28 and the fragments were detected in the Orbitrap at a resolution of 17,500. A data-dependent procedure that alternated between one MS scan followed by 20 MS/MS scans with 30.0 s dynamic exclusion. Automatic gain control (AGC) was set at 5E4.

### Database search and bioinformatics analysis

The raw MS/MS data was analyzed using MaxQuant search engine (v.1.5.2.8) and searched against swissprot Human database concatenated with reverse decoy database. Trypsin/P was specified as cleavage enzyme with maximum 2 missing cleavages. The mass tolerance for precursor ions was set as 20 ppm in First search and 5 ppm in Main search, and the mass tolerance for fragment ions was set as 0.02 Da. Carbamidomethyl on Cys was specified as fixed modification, oxidation on Met and acetylation on the protein N-terminus was specified as variable modifications. Label-free quantification method was LFQ, the false discovery rate (FDR) was adjusted to < 1% and minimum score for peptides was set > 40.

Gene Ontology (GO) analysis was conducted to annotate and classify all identified proteins based on three categories (biological process, molecular function and cell component) by the UniPort-GOA database (http://www.ebi.ac.uk/GOA/), InterPro (http://www.ebi.ac.uk/interpro/) and GO annotation (http://geneontology.org/). A cutoff of absolute fold change ≥ 1.5 was applied to identify differentially expressed proteins and P value < 0.05 was considered significant.

### Inhibition of CLEC11A (C-type lectin domain family 11, member A)

Lentivirus shRNAs targeting human CLEC11A (shCLEC11A #1, shCLEC11A #2 and shCLEC11A #3), and the scramble control shRNA (shCon) were purchased from Cyagen Biosciences. The virus packaging was completed by Cyagen Biosciences. Cell transfection was conducted according to the manufacturer's instructions. Briefly, hucMSCs were incubated in virus suspension supplemented with 5 μg/mL polybrene (Cyagen Biosciences). The culture supernatant was replaced with fresh complete medium after 8 h. 72 h later, the transfected cells were selected with puromycin (Sigma) in the medium. The sequences of shRNA used in this study were as follows: shCLEC11A #1: 5'-TGAGGACATCGTCACTTACATCTCGAGATGTAAGTGACGATGTCCTCA-3'; shCLEC11A #2: 5'-GCTAGGTCCGGTGCCAATAAACTCGAGTTTATTGGCACCGGACCTAGC-3'; shCLEC11A #3: 5'-GTGCCAAGAGTCCAGCTTAATCTCGAGATTAAGCTGGACTCTTGGCAC-3'; Con shRNA: 5'-CCTAAGGTTAAGTCGCCCTCGCTCGAGCGAGGGCGACTTAACCTTAGG-3'.

### Culture of bone marrow mesenchymal stromal cells (BMSCs) and osteoclast progenitors

BMSCs were isolated from 3-4-week-old C57BL/6 mice as previously described 30, and were cultured in α-MEM (Gibco) supplemented with 10% FBS (Gibco), 100 U/mL penicillin and 100 μg/mL streptomycin (Gibco). For mouse primary BMSC characterization, the osteogenic, adipogenic and chondrogenic differentiation media were used to evaluate the multipotency of BMSCs. The capacities of osteogenesis, adipogenesis and chondrogenesis of BMSCs were confirmed by ARS, ORO and Alcian Blue staining respectively. Flow cytometric analysis was performed to detect the expression of surface markers (CD34, CD44, CD45, CD90 and Sca-1) in BMSCs, while cell aliquots of the negative controls were incubated with isotype-matched mouse monoclonal antibodies. All antibodies were acquired from BD Biosciences. Early-passage BMSCs were used for the following experiments. The mouse osteoclast progenitor cell line RAW264.7 was purchased from American Type Culture Collection (Rockville, MD, USA) and cultured in high-glucose DMEM (Gibco) containing 15% FBS (Gibco), 100 U/mL penicillin and 100 ug/mL streptomycin (Gibco). All cells were incubated in a humidified environment at 37°C with 5% CO_2_.

### Osteogenic differentiation assay

BMSCs (1.0 × 10^5^ cells per well) were seeded into 48-well culture plates and incubated overnight. Then, osteogenesis induction medium (Cyagen Biosciences) were applied to induce osteogenic differentiation of BMSCs supplemented with or without EVs (100 μg/ml) from control hucMSCs or CLEC11A-knockdown hucMSCs. After 3 days of culture, the differentiated BMSCs were stained with an Alkaline Phosphatase (ALP) Staining Kit (YEASEN, Shanghai, China) to determine ALP activity. After 7 days of culture, quantitative real-time PCR (qRT-PCR) was performed to analyze the expression of osteogenesis-related genes. 2 weeks after osteogenic induction, the cells were assayed by 2% ARS (Solarbio, Beijing, China) staining at pH 4.2 to assess the cell matrix mineralization.

### Adipogenic differentiation assay

BMSCs (1.0 × 10^5^ cells per well) were plated in 48-well culture plates and cultured until reaching 90% confluence. Then, adipogenesis induction medium (Cyagen Biosciences) were used to induce adipogenic differentiation following the manufacturer's protocol supplemented with or without EVs (300 μg/ml) from control hucMSCs or CLEC11A-knockdown hucMSCs. After 6 days of induction, the expression of adiponectin (*Adipo*), resistin (*Retn*) and peroxisome proliferator-activated receptor-g (*Pparg*) mRNA was analyzed by qRT-PCR. After 3 weeks, the cells were stained with ORO to detect mature adipocytes.

### Osteoclast differentiation assay

RAW264.7 cells (1.0 × 10^4^ cells per well) were plated in 48-well plates and further cultured in fresh complete medium containing 100 ng/mL receptor activator for nuclear factor-κB ligand (RANKL; Peprotech, London, England) supplemented with an equal volume of PBS or hucMSC-EVs (100 μg/ml) from control hucMSCs or CLEC11A-knockdown hucMSCs. qRT-PCR was conducted to examine the expression levels of osteoclastogenesis-related genes at day 5 of induction. After 8 days of culture, the cells were fixed and stained for TRAP with a commercially kit (Sigma) to detect TRAP activity of osteoclasts. TRAP-positive cells with more than three nuclei were regarded as mature osteoclasts. The number of osteoclasts was counted by an inverted microscopy (Leica).

### qRT-PCR analysis

Total RNA from cultured cells was extracted using Trizol (Invitrogen, Carlsbad, USA) and cDNA was synthesized from equal quantities of RNA using a Revert Aid First-strand cDNA Synthesis Kit (Fermentas, Burlington, Canada). Then, equal volumes of cDNA were amplified in an ABI PRISM® 7900HT System (Applied Biosystems, Foster City, USA) with FastStart Universal SYBR Premix ExTaq^TM^II (Takara Biotechnology, Dalian, China). Relative mRNA expression was calculated by the relative standard curve method (2^-△△CT^) using GAPDH as the internal control. The primers used for qPCR analysis were as follows: *Runx2* (runt-related transcription factor 2): forward, 5'-GACTGTGGTTACCGTCATGGC-3', and reverse, 5'-ACTTGGTTTTTCATAACAGCGGA-3'; *Sp7* (osterix): forward, 5'-ATGGCGTCCTCTCTGCTTGA-3', and reverse, 5'-GAAGGGTGGGTAGTCATTTG-3'; *Col1a1* (collagen type I α1): forward, 5'-GACATGTTCAGCTTTGTGGACCTC-3', and reverse, 5'-GGGACCCTTAGGCCATTGTGTA-3'; *Bglap* (OCN): forward, 5'-CTGACCTCACAGATC CCAAGC-3', and reverse, 5'-TGGTCTGATAGCTCGTCACAAG-3'; *Dmp1* (dentin matrix protein 1): forward, 5'-TGGGAGCCAGAGAGGGTAG-3', and reverse, 5'-TTGTGGTATCTGGCAACTGG-3'; *Tnfsf11* (RANKL): forward, 5'-GCCATTTGCACACCTCACCA-3', and reverse, 5'-GCCGAAAGCAAATGTTGGCG-3'; *Adipo*: forward, 5'-GATGGCAGAGATGGCACTCC-3', and reverse, 5'-CTTGCCAGTGCTGCCGTCAT-3'; *Retn*: forward, 5'-TTGCTGGACAGTCTCCTCCAGAGGG-3', and reverse, 5'-AAGCGACCTGCAGCTTACAGCAG-3'; *Pparg*: forward, 5'-TGTCTCATAATGCCATCAGGTTTG-3', and reverse, 5'-GATAACGAATGGTGATTTGTCTGTT-3'; *Nfatc1* (nuclear factor of activated T cells c1): forward, 5'-CAGTGTGACCGAAGATACCTGG-3', and reverse, 5'-TCGAGACTTGATAGGGACCCC-3'; *Trap*: forward, 5'-TGGTCCAGGAGCTTAACTGC-3', and reverse, 5'-GTCAGGAGTGGGAGCCATATG-3'; *Ctsk* (cathepsin K): forward, 5'-GCGGCATTACCAACAT-3', and reverse, 5'-CTGGAAGCACCAACGA-3'; *Gapdh*: forward, 5'-CACCATGGAGAAGGCCGGGG-3', and reverse, 5'-GACGGACACATTGGGGGTAG-3'; *CLEC11A*: forward, 5'-CTGCCGGAACTGTTGAGGG-3', and reverse, 5'-CCCAGGATGTAAGTGACGATGT-3'; *GAPDH*: forward, 5'-GACCACAGTCCATGCCATCAC-3', and reverse, 5'-TCCACCACCCTGTTGCTGTAG-3'.

### Western Blot analysis

Total proteins of cells or EVs were extracted using RIPA lysis buffer containing a protease inhibitor cocktail (Sigma). Extracted proteins were subjected to SDS-PAGE seperation and further transferred onto polyvinylidene fluoride membranes (Millipore). The membranes were incubated with primary antibodies overnight at 4°C after blocking with 5% non-fat milk. Then, the membranes were washed and incubated with horseradish peroxidase-conjugated secondary antibodies for 1 h at room temperature. Blots were developed using an enhanced chemiluminescence reagent (Thermo) and visualized by the ChemiDoc^TM^ XRS Plus luminescent image analyser (Bio-Rad). Primary antibodies were used in this study as follows: anti-CD9 (1:500; Santa Cruz, Dallas, USA), anti-CD63 (1:500; Santa Cruz), anti-CD81 (1:500; Santa Cruz), anti-TSG101 (1:1000; ProteinTech, Chicago, USA), anti-CLEC11A (1:2000; R&D Systems, Minneapolis, USA) and anti-GAPDH (1:5000; Cell Signaling Technology, Danvers, USA). All the secondary antibodies (1:5000) were acquired from Cell Signaling Technology.

### Statistical analysis

Data are presented as means ± standard deviation (SD). Two-tailed Student's *t* test was used to compare means between two groups. Statistical analysis was performed using GraphPad Prism software and differences were considered statistically significant at *P* < 0.05.

## Results

### Characterization of hucMSCs and hucMSC-EVs

**Figure 1:**
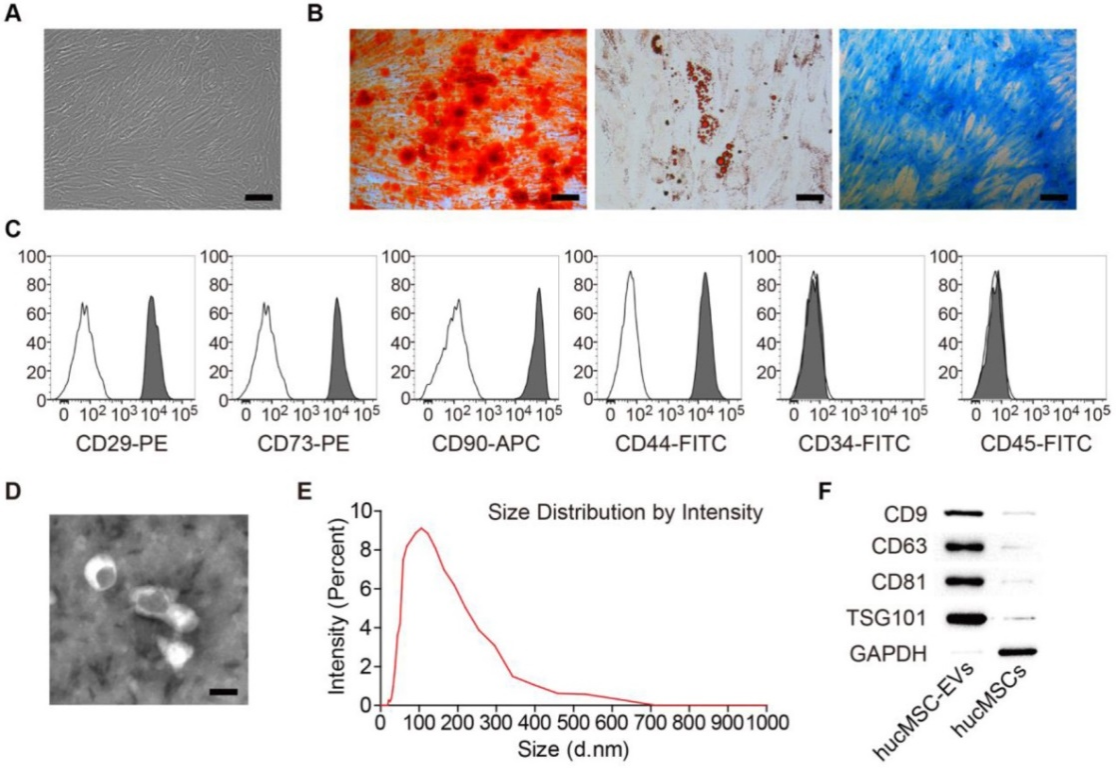
** Characterization of hucMSCs and hucMSC-EVs. (A)** hucMSCs showed a spindle fibroblast-like morphology. Scale bar: 100 μm. **(B)** hucMSCs were capable of differentiating into osteoblasts, adipocytes or chondrocytes after osteogenic, adipogenic or chondrogenic medium induction, indicated by Alizarin Red S (ARS) staining, Oil Red O (ORO) staining and Alcian Blue staining. Scale bars: 100 μm (left); 50 μm (middle); 100 μm (right). **(C)** Flow cytometry analysis of the typical surface markers in hucMSCs. Blank curves: the isotype controls; solid gray curves: the test samples. **(D)** Morphology of hucMSC-EVs under transmission electron microscopy. Scale bar: 100 nm. **(E)** Size distribution of hucMSC-EVs calculated by dynamic light scattering analysis. **(F)** Detection of the EV surface markers (CD9, CD63, CD81 and TSG101) in hucMSC-EVs by Western Blot.

MSCs isolated from human umbilical cord exhibited a spindle fibroblast-like morphology (**Figure 1A**) and were able to differentiate into osteoblasts, adipocytes or chondrocytes after osteogenic, adipogenic or chondrogenic medium induction (**Figure 1B**). Flow cytometric analysis showed that hucMSCs positively expressed CD29, CD44, CD73 and CD90, but negatively expressed CD34 and CD45 (**Figure 1C**). The obtained cells had the typical characteristics of MSCs and were consistent with the previous reports 33,34. TEM, dynamic light scattering analysis and Western Blot analyses were performed to characterize the EVs derived from hucMSCs. hucMSC-EVs presented a cup or sphere-shaped morphology and their diameters primarily ranged from 60 nm to 150 nm (**Figure 1D-E**). Western Blot analysis demonstrated that these nanovesicles expressed exosomal marker proteins including CD9, CD63, CD81 and TSG101 (**Figure 1F**). The data indicate that these nanoparticles are EVs.

### hucMSC-EVs prevent osteoporosis by maintaining bone mass and strength

**Figure 2:**
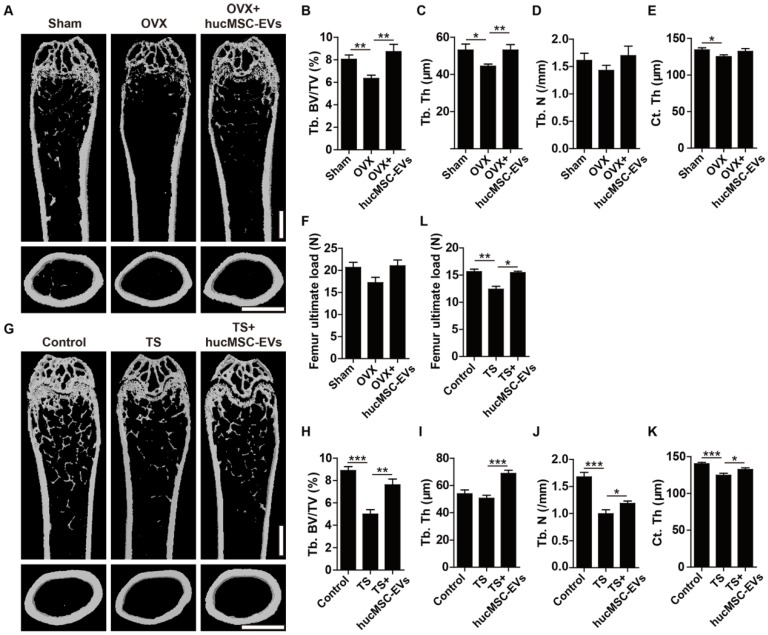
** hucMSC-EVs increase bone mass and strength in OVX- and TS-induced osteoporotic mice. (A)** Representative μCT images of femora from sham-operated, OVX and OVX treated with hucMSC-EVs mice. Scale bar: 1 mm. **(B-E)** Quantification of trabecular and cortical bone microarchitecture. n = 8 per group for OVX + hucMSC-EVs; n = 10 for other groups. Tb. BV/TV: trabecular bone fraction; Tb. Th: trabecular thickness; Tb. N: trabecular number; Ct. Th: cortical thickness. **(F)** Three-point bending measurement of femur maximum load. n = 5 per group.** (G-K)** Representative μCT images** (G)** and quantitative analysis of Tb. BV/TV **(H)**, Tb. Th **(I)**, Tb. N **(J)** and Ct. Th **(K)** in femora from Control, TS and TS treated with hucMSC-EVs mice. Scale bar: 1 mm. n = 10 per group for Control; n = 9 for other groups.** (L)** Detection of femur maximum load by three-point bending measurement. n = 5 per group. *P < 0.05, **P < 0.01, ***P < 0.001.

To explore the effect of hucMSC-EVs on primary and secondary osteoporosis, we established animal models of OVX-induced postmenopausal osteoporosis, senile osteoporosis and TS-induced hindlimb disuse osteoporosis, respectively. hucMSC-EVs or an equal volume of vehicle (PBS) were intravenously administrated to the three osteoporotic models. For mouse model of postmenopausal osteoporosis, the size and weight of uterus in OVX mice were significantly decreased compared to the control Sham mice (**Figure S1A-B**), which supported the success of OVX. μCT scanning revealed that PBS-treated OVX mice had reduced trabecular and cortical bone mass in the femur relative to Sham mice, whereas the intravenous administration of hucMSC-EVs to OVX mice for 2 months alleviated bone loss (**Figure 2A**). Quantification of bone microstructural parameters showed that the Tb. BV/TV, Tb. Th and Ct. Th in PBS-treated OVX mice were significantly lower than those in Sham group, while all these down-regulated parameters induced by OVX were reversed after hucMSC-EVs treatment (**Figure 2B-E**). Three-point bending test demonstrated that value of the femur maximum load (representation of bone strength) in OVX mice was lower compared with Sham mice, and the reduction of bone strength was rescued by hucMSC-EVs in some degree, but the above trend had no statistically significance (**Figure 2F**).

For mouse models of disuse osteoporosis, 3-week TS was performed to make the hindlimb of mice unloading. μCT analysis showed that the femurs of TS mice had obvious bone loss phenotypes (**Figure 2G**), as indicated by significantly lower Tb. BV/TV, Tb. N, and Ct. Th relative to the hindlimb-loading mice (**Figure 2H-K**). Consistent with the results of postmenopausal osteoporosis model, systemic injection of hucMSC-EVs twice a week for 3 weeks remarkably improved trabecular and cortical bone mass in hindlimb unloading-induced osteoporotic mice (**Figure 2G**), including significantly higher Tb. BV/TV, Tb. Th, Tb. N, and Ct. Th relative to PBS-treated TS mice (**Figure 2H-K**). Three-point bending test revealed that bone strength was also significantly enhanced in EVs-treated TS mice compared to the control TS mice (**Figure 2L**). For mouse model of senile osteoporosis, we detected that hucMSC-EVs injection enhanced trabecular bone mass of the distal femur in old mice compared with the PBS-treated control mice (**Figure S2A-C**). However, the difference between the EVs-treated group and control group was not significant. All these findings suggest that hucMSC-EVs are capable of preventing the loss of bone mass and strength in primary and secondary osteoporotic mice.

### hucMSC-EVs increase bone formation, reduce marrow fat accumulation and inhibit osteoclastogenesis

**Figure 3:**
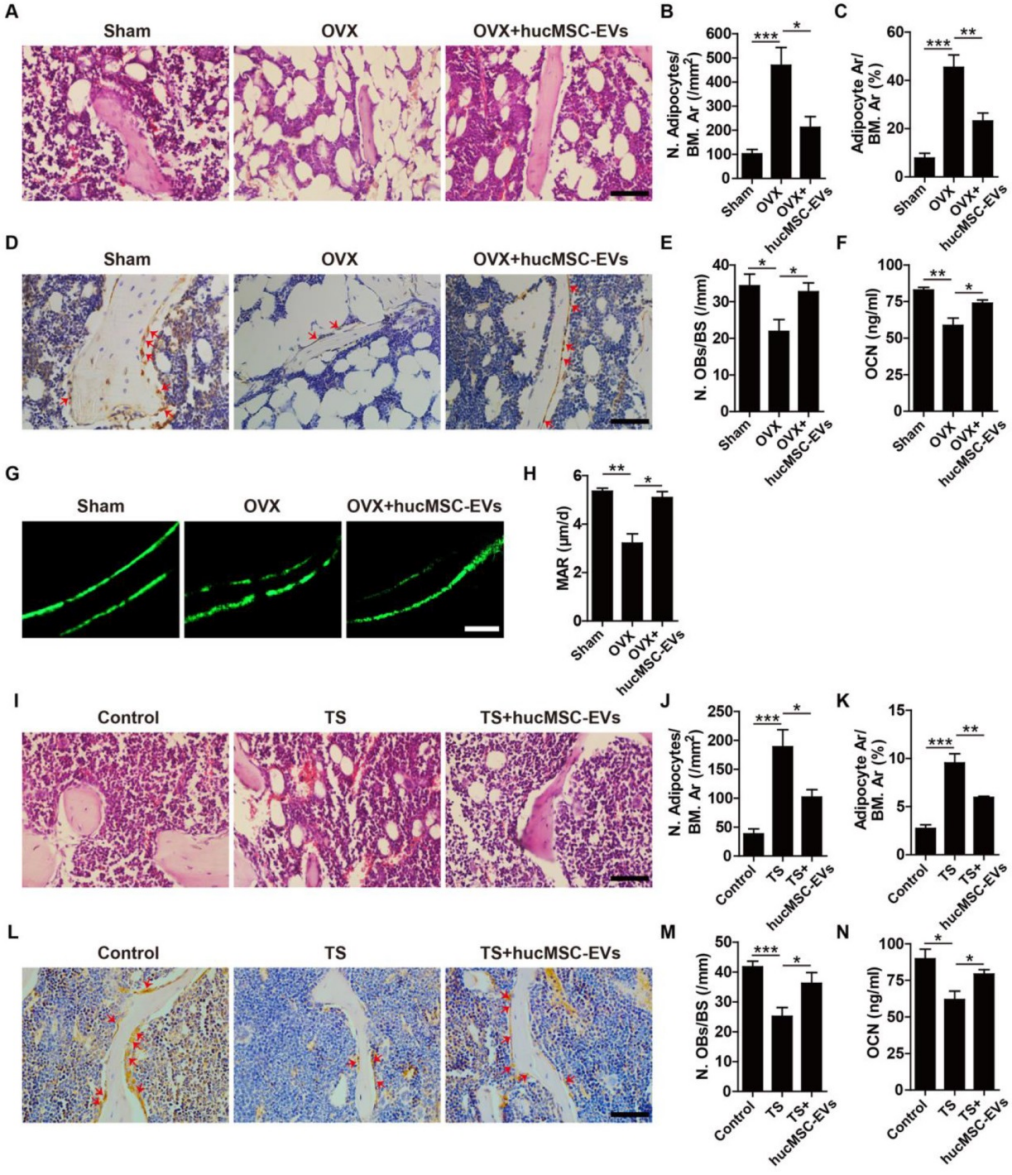
** hucMSC-EVs enhance bone formation and reduce marrow fat accumulation. (A)** Representative images of H&E staining in distal femoral metaphysis from Sham, OVX and OVX + hucMSC-EVs mice. Scale bar: 50 μm. **(B-C)** Quantitative analysis of the number (N.) and area (Ar) of adipocytes in **(A)**. n = 3 per group. BM: bone marrow. **(D)** Representative OCN immunostaining images of distal femora in Sham, OVX and OVX + hucMSC-EVs mice. Red arrows indicate osteoblasts (OBs). Scale bar: 50 μm. (**E**) Quantitative analysis of the number of OBs of trabecular bone surface (BS) in **(D)**. n = 3 per group. **(F)** The serum levels of OCN in Sham, OVX and OVX + hucMSC-EVs mice detected by ELISA. n = 4 per group for OVX; n = 5 for other groups. **(G-H)** Representative images of calcein double labeling **(G)** and quantification of mineral apposition rate (MAR; **H**) in trabecular bone of femora. Scale bar: 25 μm. n = 3 per group. **(I-M)** Representative H&E staining **(I)** and OCN immunostaining images **(L)** with quantitative analysis of the number and area of adipocytes **(J-K)** and the number of OBs **(M)** in distal femora from Control, TS and TS + hucMSC-EVs mice. Scale bar: 50 μm. n = 3 per group. **(N)** The OCN serum levels in Control, TS and TS + hucMSC-EVs mice detected by ELISA. n = 5 per group. *P < 0.05, **P < 0.01, ***P < 0.001.

To investigate the mechanism by which hucMSC-EVs exert bone-sparing effect *in vivo*, we used multiple approaches to determine whether they could regulate osteogenesis in OVX- and TS-induced osteoporotic mice. H&E staining of femurs revealed that the number and area of adipocytes featured by fat vacuoles in bone marrow of OVX mice were significantly increased relative to Sham mice, whereas hucMSC-EVs treatment significantly reduced the accumulation of fat cells (**Figure 3A-C**). Immunohistochemical staining for OCN (a bone formation marker) showed that PBS-treated OVX mice had significantly lower number of OCN-positive osteoblasts on the trabecular bone surface compared with Sham mice, but the number of osteoblasts was significantly increased after hucMSC-EVs administration (**Figure 3D-E**). The concentration of OCN protein in serum determined by ELISA also significantly increased when OVX mice were administered with hucMSC-EVs (**Figure 3F**). Calcein double labeling was performed to further confirm that EVs-treated OVX mice had significantly higher MAR, which represents the capacity of new bone mineralization, compared to their control OVX mice (**Figure 3G-H**). Consistent with the above results, histological, immunohistochemical and ELISA analyses of samples from TS mouse model revealed that TS-operation led to significantly increased bone marrow fat accumulation, and significantly decreased osteoblasts number of the trabecular bone surface and OCN secretion compared to the control mice, while all these changes were rescued by intravenous injection of hucMSC-EVs (**Figure 3I-N**). The functions of hucMSC-EVs that decrease marrow fat accumulation and increase the number of osteoblasts imply its promotion on the switch from adipogenic differentiation to osteogenic differentiation of BMSCs.

**Figure 4:**
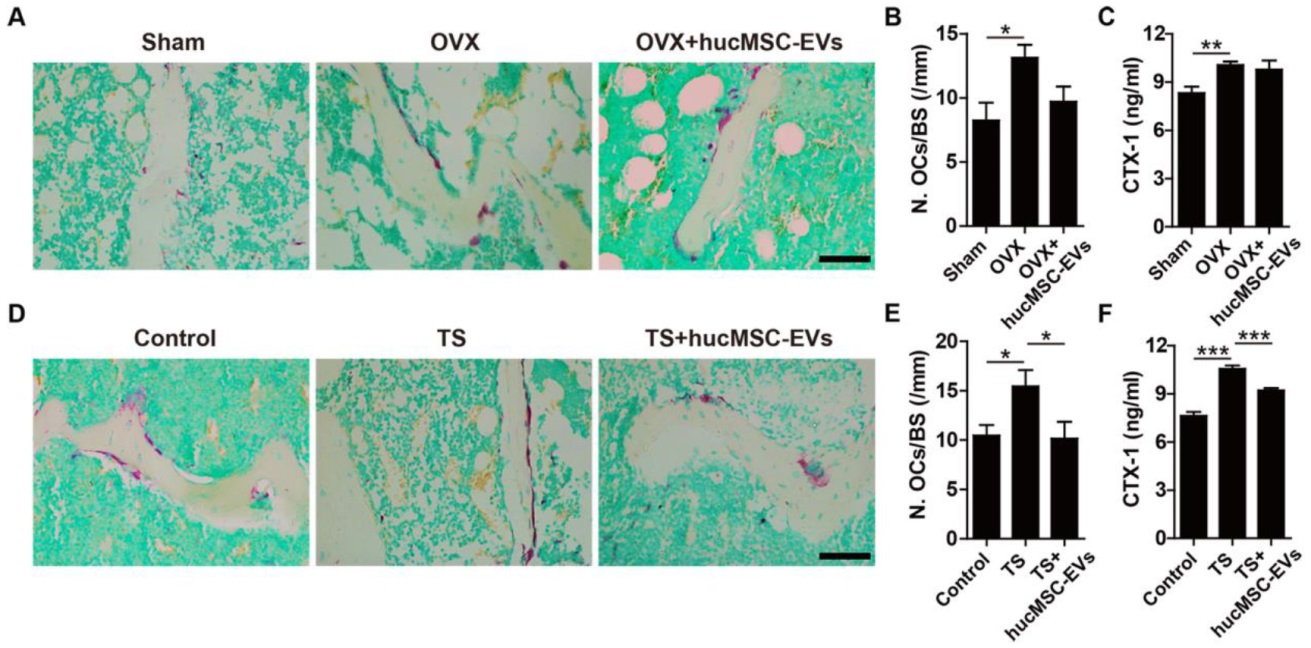
**hucMSC-EVs inhibit osteoclast formation. (A)** Representative images of TRAP staining in distal femora from Sham, OVX and OVX + hucMSC-EVs mice. Scale bar: 50 μm. **(B)** Quantification of the number of osteoclasts (OCs) on trabecular bone surface in (A). n = 3 per group. **(C)** The serum levels of CTX-I in Sham, OVX and OVX + hucMSC-EVs mice detected by ELISA. n = 4 per group for OVX; n = 5 for other groups. **(D-E)** TRAP staining of femora **(D)** from Control, TS and TS + hucMSC-EVs mice with quantification of the number of OCs **(E)**. Scale bar: 50 μm. n = 3 per group. **(F)** The CTX-I serum levels in Control, TS and TS + hucMSC-EVs mice detected by ELISA. n = 5 per group. *P < 0.05, **P < 0.01, ***P < 0.001.

Osteogenesis is coupled with osteoclastogenesis during bone remodeling. Thus, we next evaluated the effect of hucMSC-EVs on osteoclast formation. TRAP staining of femur sections was used to examine osteoclasts. As shown in **Figure 4A-B, D-E**, the quantity of osteoclasts on the trabecular bone surface in mice that underwent OVX or TS was significantly larger than that in their respective control mice, whereas hucMSC-EVs treatment could inhibit osteoclast formation. The difference in the number of osteoclasts was statistically significant in TS mice, but not in OVX mice. Consistently, ELISA analysis demonstrated that the serum level of CTX-I (a bone resorption marker) was significantly up-regulated in TS mice compared to the control mice, but the up-regulation was remarkably reversed by hucMSC-EVs (**Figure 4F**). However, no significant difference was observed in the concentration of CTX-I between the PBS- and EVs-treated OVX mice (**Figure 4C**).

These *in vivo* results suggest that hucMSC-EVs can restore the balance of bone metabolism in osteoporotic mice by enhancing osteoblast formation and inhibiting adipocyte and osteoclast formation.

### Proteomic analysis of hucMSCs and hucMSC-EVs

**Figure 5:**
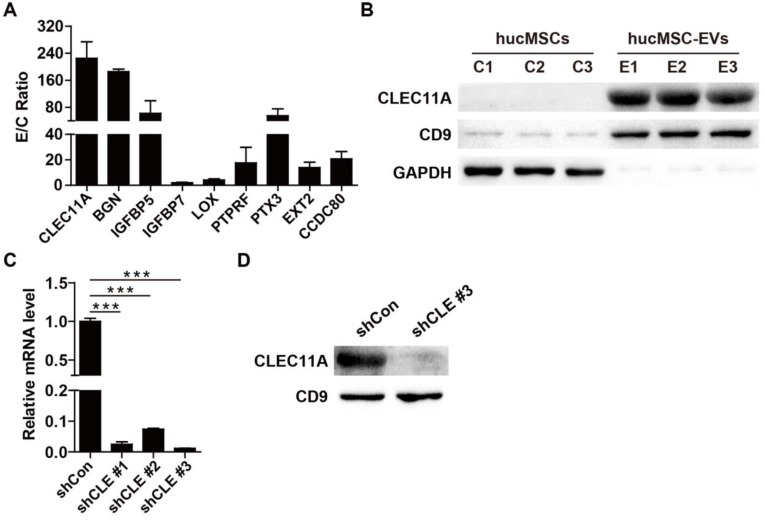
** Proteomic analysis of hucMSCs and hucMSC-EVs. (A)** The expression ratio of a class of bone-protective proteins in hucMSC-EVs compared to that in hucMSCs. n = 3 per group. **(B)** The protein level of CLEC11A in hucMSC-EVs and hucMSCs was verified by Western Blot. n = 3 per group. **(C)** The silenced efficiency of shRNAs targeting CLEC11A was evaluated by qRT-PCR analysis. shCon: shControl; shCLE: shCLEC11A. n = 3 per group. **(D)** The absence of CLEC11A in hucMSC-EVs was verified by Western Blot analysis. *P < 0.05, **P < 0.01, ***P < 0.001.

To detect the critical molecules that mediate the functions of hucMSC-EVs, proteomic analysis was performed to test the protein profiles of hucMSC-EVs and their parent cell hucMSCs. The label-free LC-MS/MS analysis totally identified 5570 proteins, among which 4615 proteins was quantified. All identified proteins in hucMSC-EVs and hucMSCs with quantitative information are listed in **Table S1**. GO analysis was used to annotate and classify all identified proteins based on three categories, including biological process, molecular function and cell component. Statistical analysis of differentially expressed proteins revealed that the expression of 808 proteins was significantly different between hucMSC-EVs and hucMSCs with ≥ 1.5 fold change and *P* value < 0.05. Among them, 61 proteins in hucMSC-EVs were significantly up-regulated and 747 proteins were significantly down-regulated relative to their parent cell hucMSCs (**Table S1**). The upregulated proteins were further biologically interpreted to explore their involved biological processes or function. The results showed that hucMSC-EVs were highly enriched in the proteins related to bone growth and development (**Figure 5A**). The expression ratio of a class of pro-osteogenic and/or anti-adipogenic proteins in hucMSC-EVs compared with that in hucMSCs are shown in **Figure 5A**. The data indicated that CLEC11A, which has been reported to be a new osteogenic factor and potently promote osteogenesis, was upregulated with the highest E/C ratio (224.86 ± 84.36-fold) among all the hucMSC-EVs-enriched proteins (**Figure 5A**). Thus, we selected CLEC11A as the candidate protein to study the molecular mechanism by which hucMSC-EVs exert the preventive effect of bone loss. The high enrichment of CLEC11A in hucMSC-EVs relative to hucMSCs was confirmed by Western Blot (**Figure 5B**), which was consistent with the proteomic result.

### Silencing of *CLEC11A* in hucMSCs

Then, we used three shRNAs (shCLEC11A #1, shCLEC11A #2 and shCLEC11A #3) to silence the expression of *CLEC11A* in hucMSCs by cell transfection and the inhibitory efficiency of the three shRNAs was evaluated by qRT-PCR (**Figure 5C**). The results showed that shCLEC11A #3 had the highest inhibitory efficiency (**Figure 5C**). The culture supernatants from hucMSCs transfected with the shCLEC11A #3 or the shCon were collected to extract EVs for the downstream experiments. The down-regulation of CLEC11A in EVs from CLEC11A-silenced hucMSCs (hucMSC^shCLEC11A #3^-EVs) relative to hucMSC^shCon^-EVs was verified through Western Blot analysis (**Figure 5D**).

### hucMSC-EVs promote osteogenic differentiation and inhibit adipogenic differentiation of BMSCs by transferring CLEC11A

**Figure 6:**
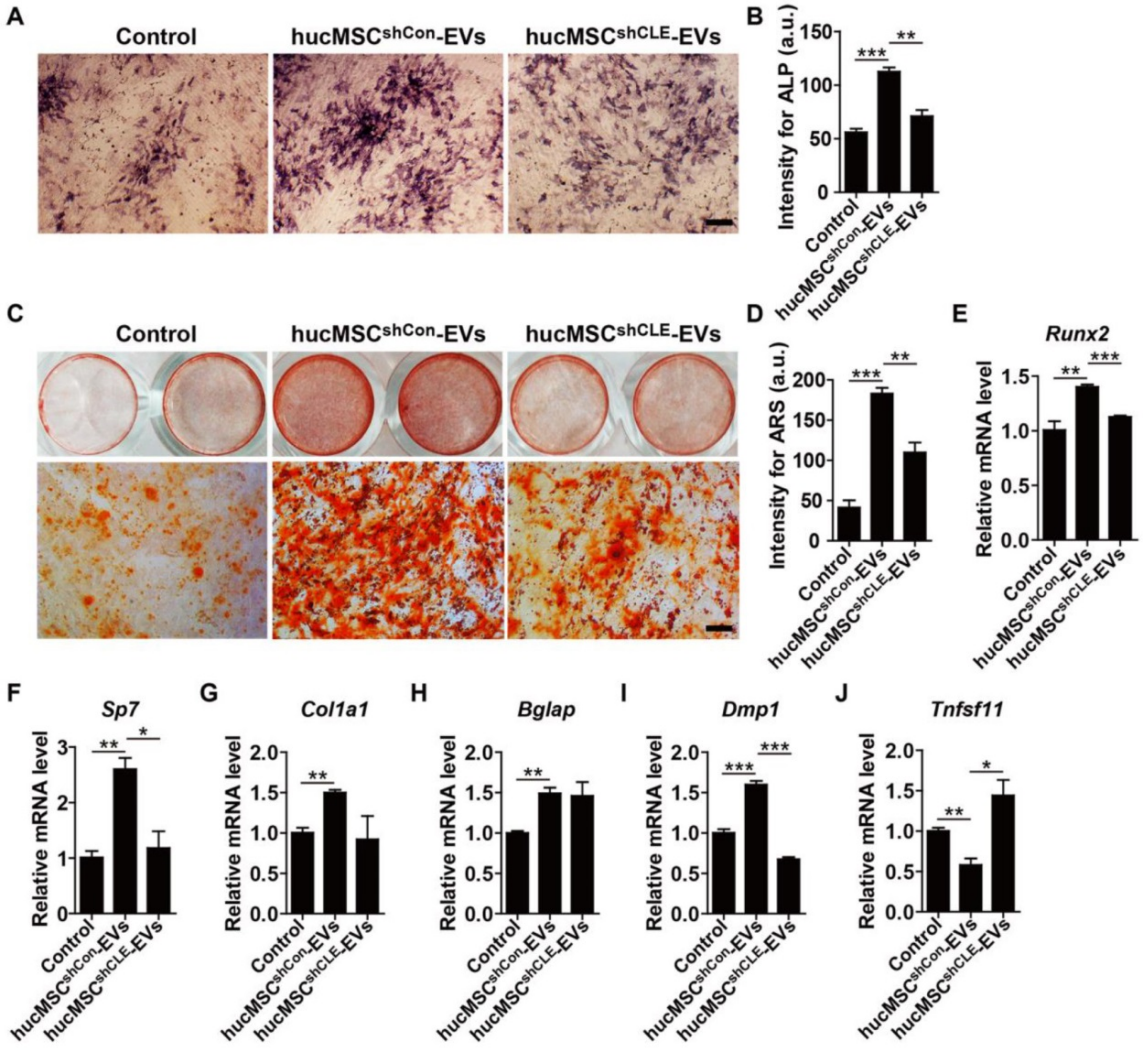
** hucMSC-EVs stimulate osteogenic differentiation of BMSCs by transferring CLEC11A.** (**A-D**) Representative images of Alkaline Phosphatase (ALP) staining **(A)** and ARS staining **(C)** and quantification of the intensity for staining** (B, D)** in BMSCs treated with PBS and EVs from CLEC11A-silenced hucMSCs (hucMSCs^shCLE^-EVs) or shCon-treated hucMSCs (hucMSCs^shCon^-EVs) under osteogenic culture condition. Scale bar: 100 μm (ALP); 50 μm (ARS). n = 3 per group. **(E-J)** qRT-PCR analysis of the expression levels of *Runx2, Sp7, Col1a1, Bglap, Dmp1* and *Tnfsf11* in BMSCs at day 7 of induction. n = 3 per group. *P < 0.05, **P < 0.01, ***P < 0.001.

To determine whether hucMSC-EVs promote osteogenesis and/or inhibit adipogenesis *in vitro* and the role of CLEC11A in these processes. We firstly isolated primary BMSCs and characterized them for MSCs (**Figure S3A-C**). Then, BMSCs were induced to differentiate into osteoblast under osteogenic culture conditions and treated with hucMSCs^shCon^-EVs, hucMSCs^shCLEC11A #3^-EVs or an equal volume of PBS. As shown in **Figure 6A-D**, hucMSC-EVs significantly enhanced osteoblast differentiation of BMSCs, as evidenced by the increased ALP activity and calcium mineral deposition in the hucMSCs^shCon^-EVs-treated group compared to the PBS-treated group, while silencing of CLEC11A in the parent cell hucMSCs remarkably abolished the positive effects of hucMSC-EVs. qRT-PCR analysis showed that the mRNA levels of osteoblast differentiation markers (*Runx2, Sp7, Col1a1, Bglap* and *Dmp1*) were significantly up-regulated after hucMSC-EVs treatment, whereas the pro-osteoblastic function of EVs was partially inhibited when CLEC11A content was reduced in hucMSC-EVs (**Figure 6E-I**). We also tested the expression of *Tnfsf11*, a critical mediator of osteoclast differentiation. The result revealed a significantly decreased expression level of *Tnfsf11* mRNA in the differentiated BMSCs treated with hucMSCs^shCon^-EVs, suggesting that hucMSC-EVs may inhibit osteoclastic activity by reducing RANKL production in osteoblasts. Of note with regard to our study, hucMSCs^shCLEC11A #3^-EVs did not exhibit this effect (**Figure 6J**).

**Figure 7:**
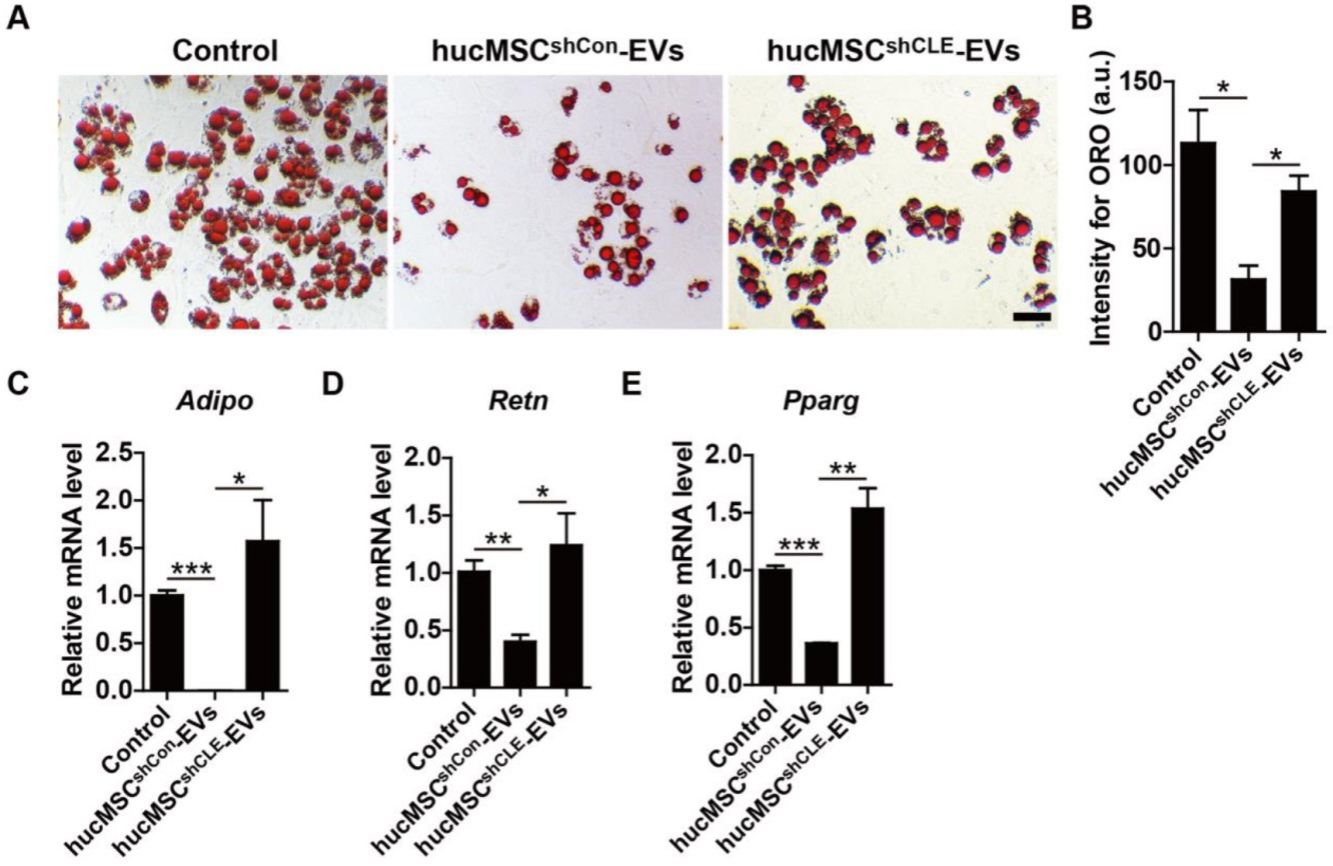
**hucMSC-EVs inhibit adipogenic differentiation of BMSCs via delivering CLEC11A. (A)** Representative ORO staining images of BMSCs treated with PBS, hucMSCs^shCon^-EVs and hucMSCs^shCLE^-EVs under adipogenic culture condition. Scale bar: 50 μm. **(B)** Quantification of the intensity for ORO staining in **(A)**. n = 3 per group. **(C-E)** qRT-PCR analysis of the expression levels of *Adipo*, *Retn* and *Pparg* at day 6 of induction. n = 3 per group. *P < 0.05, **P < 0.01, ***P < 0.001.

In addition, BMSCs were cultured in adipogenic induction media supplemented with hucMSCs^shCon^-EVs, hucMSCs^shCLEC11A #3^-EVs or an equal volume of PBS. The ORO staining and qRT-PCR data showed that hucMSC-EVs significantly inhibited lipid droplet formation of BMSCs induced by adipogenic induction media (**Figure 7A-B**), accompanied by down-regulated mRNA levels of *Adipo, Retn* and *Pparg*, several key markers of adipocyte differentiation (**Figure 7C-E**). In contrast, hucMSCs^shCLEC11A #3^-EVs had weaker ability to attenuate adipogenic differentiation of BMSCs and decrease the expression of adipogenesis-related genes in BMSCs (**Figure 7A-E**). All of these results demonstrate that hucMSC-EVs promote osteogenic differentiation and inhibit adipogenic differentiation of BMSCs *in vitro*, and CLEC11A mediates the beneficial effects.

### CLEC11A is required for the anti-osteoclastic effects of hucMSC-EVs

**Figure 8:**
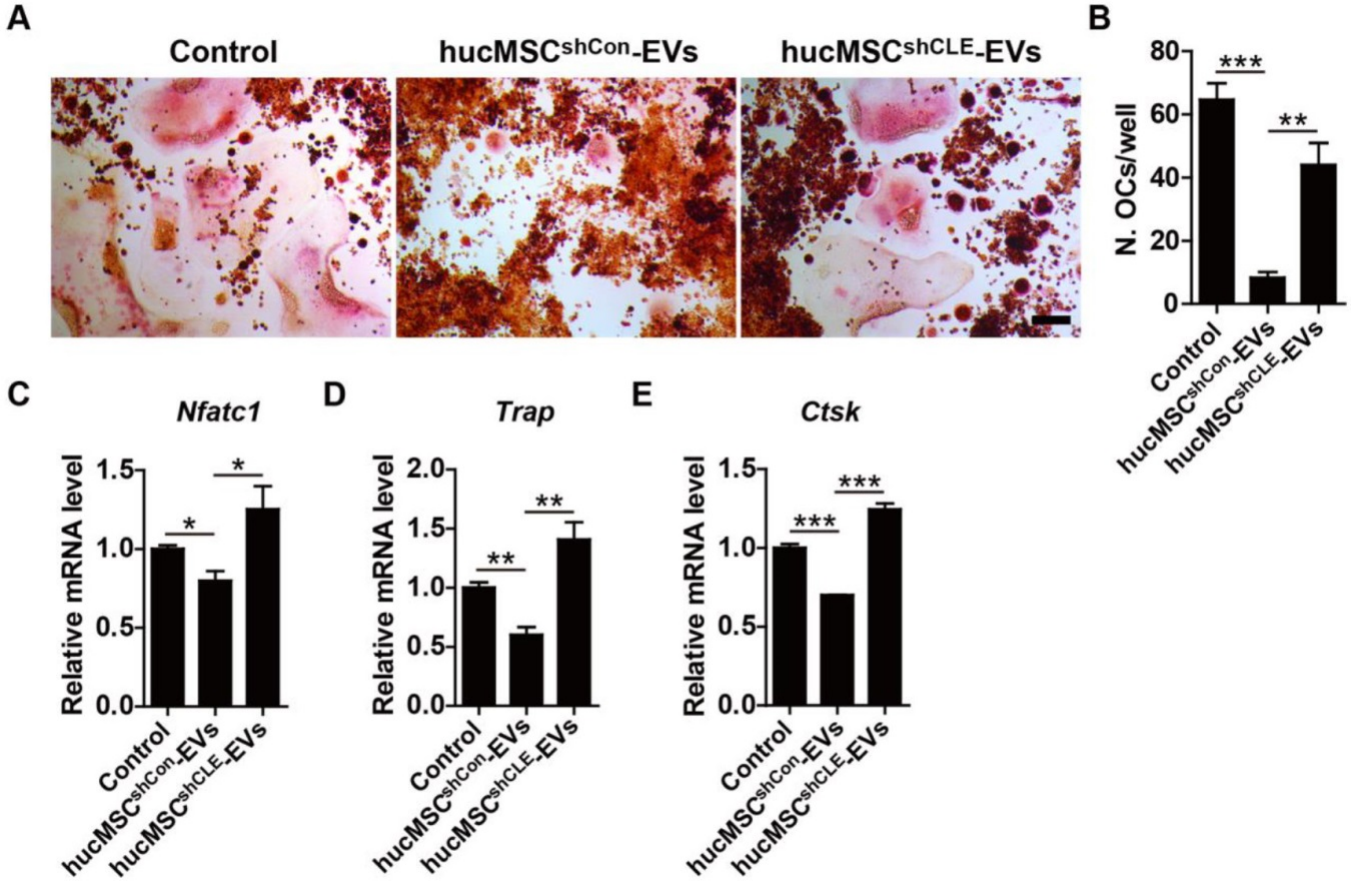
**CLEC11A is required for the anti-osteoclastic effects of hucMSC-EVs. (A)** TRAP staining of RAW264.7 cells cultured in RANKL-containing medium treated with PBS, hucMSCs^shCon^-EVs and hucMSCs^shCLE^-EVs. Scale bar: 100 μm. **(B)** Quantitative analysis of the number of TRAP positive OCs in **(A)**. n = 3 per group. **(C-E)** qRT-PCR analysis of the expression levels of *Nfatc1*, *Trap* and *Ctsk* at day 5 of induction. n = 3 per group. *P < 0.05, **P < 0.01, ***P < 0.001.

Finally, we assessed the direct impact of hucMSC-EVs on osteoclast formation and the role of CLEC11A during this process. RAW264.7 cells were induced to differentiate into osteoclasts by RANKL and simultaneously received the above different treatments. As revealed by TRAP staining, hucMSC-EVs significantly suppressed the formation of mature osteoclasts, while the anti-osteoclastic ability was lower in the hucMSCs^shCLEC11A #3^-EVs group compared to hucMSCs^shCon^-EVs group (**Figure 8A-B**). qRT-PCR was also conducted to detect the gene expression of osteoclast differentiation markers including *Nfatc1, Trap* and *Ctsk*. The results indicated that the mRNA levels of these genes were significantly decreased by hucMSC-EVs in the differentiated RAW264.7 cells (**Figure 8C-E**). Notably, the EVs-induced down-regulation of osteoclastogenesis-related genes was reversed when CLEC11A was knocked down in their parent cells. These data suggest that CLEC11A is required for the inhibition of hucMSC-EVs on osteoclastic formation.

## Discussion

In the present study, we found that hucMSC-EVs, the nanocarriers naturally secreted by hucMSCs, could effectively prevent bone loss and maintain bone strength in osteoporotic mice through multiple actions of regulating bone metabolism, including enhancing bone formation, reducing marrow fat accumulation and decreasing bone resorption. Strikingly, we demonstrated that CLEC11A, a potently pro-osteogenic protein, was extremely highly enriched in hucMSC-EVs. *In vitro*, we showed that hucMSC-EVs could promote the shift from adipogenic to osteogenic differentiation of BMSCs via delivering CLEC11A. Furthermore, CLEC11A was also required for the inhibitory effects of hucMSC-EVs on osteoclast formation.

Over the past decades, the application of MSCs for bone tissue repair and regeneration has received considerable attention 13,35,36. Several studies have reported that the paracrine actions are the main mechanism by which MSCs exert their therapeutic effects 11,12. EVs, as important paracrine factors that mediate the intercellular communication, are critical effectors of MSCs 37-40. MSC-derived EVs have therapeutic effects in variety of diseases including bone defect repair and fracture healing 11,41-43, due to their specific advantages of high stability, low immunogenicity, non-tumorigenicity and non-vascular thrombosis 39,44. Thus, direct use of MSCs-derived EVs may be a safer and promising therapeutic strategy for bone regeneration. hucMSC-EVs have been utilized to treat various disease models such as myocardial infarction 45, liver fibrosis 26, cutaneous wound 46 and bone fracture healing 25. However, the roles of hucMSC-EVs in systemic osteoporosis remain unclear. In this study, we generated mouse models of OVX-, aging- and TS-induced osteoporosis and investigated the influence of hucMSC-EVs on primary and secondary osteoporosis by intravenous injection. Based on μCT and biomechanical analysis, we found that hucMSC-EVs significantly increased bone mass and strength in OVX- and TS-induced osteoporotic mice. Our previous work revealed that human umbilical cord blood-derived EVs could ameliorate senile osteoporosis through maintaining bone mass 30. Nevertheless, the volume of umbilical cord blood is relatively limited and EVs acquired from them are not too enough for large-scale clinical application. Thus, EVs isolated from hucMSCs, which possess powerful self-renewal and proliferation capacity *in vitro*, may represent a preferable agent for treating osteoporosis.

It is well known that bone resorption is coupled with bone formation during bone remodeling, while osteoblast formation is closely related with adipocyte formation in the bone marrow milieu 2,3. Either uncoupled bone remodeling or unbalanced osteogenic and adipogenic differentiation of BMSCs leads to bone loss and osteoporosis 47. Our study showed that hucMSC-EVs administration not only promoted bone formation but also decreased bone marrow fat accumulation in osteoporotic mice, as evidenced by increased osteoblast numbers and serum level of OCN, and consequently enhanced new bone mineralization capacity as well as reduced number and area of adipocytes, suggesting that hucMSC-EVs can regulate the differentiation fate of BMSCs from adipocyte to osteoblast. In addition, hucMSC-EVs also inhibited bone resorption *in vivo*, indicated by decreased number of osteoclasts and serum level of CTX-I. However, the impact of hucMSC-EVs on osteoclast formation was not as significant as their effects on osteogenensis and adipogenesis, which might be attributed to the coupled increase in bone resorption in response to the promotion of osteogenensis induced by hucMSC-EVs. Consistently, our data from *in vitro* experiments further confirmed the *in vivo* results. The above findings indicate that the regulation of endogenous BMSCs and osteoclasts may be the key mechanisms through which hucMSC-EVs exert bone-protective effect.

EVs can regulate the function properties of specific cells by transferring bioactive proteins and RNAs from their original cells 48. Thus, we further explored the functional molecules of hucMSC-EVs by proteomic analysis. CLEC11A, also called stem cell growth factor, is a secreted sulfated glycoprotein highly expressed in the bone marrow and has long been regarded as a hematopoietic growth factor over the past decades 49-51. Recent studies have demonstrated that CLEC11A is a powerful osteogenic factor 52,53. Yue *et al*. reported that CLEC11A can be expressed by leptin-receptor^+^ (Lepr^+^) BMSCs, osteoblasts and osteocytes, and is necessary for normal adult skeleton maintenance; CLEC11A can potently promote bone formation by stimulating mesenchymal progenitors to differentiate into mature osteoblasts *in vivo* and *in vitro* 52. Shen *et al*. verified that CLEC11A exert osteogenic activity by binding to the receptor integrin α11 (Itga11) expressed by Lepr+ cells and osteoblasts, and *Itga11* conditional knockout mice exhibit decreased osteogenesis and increased bone loss during adulthood similar to *CLEC11A* deficient mice 53. In our study, data of the proteomics and Western Blot showed that CLEC11A was also expressed by hucMSCs and extremely highly enriched in hucMSC-EVs. The modulation effects of hucMSC-EVs on the differentiation directions of BMSCs and osteoclast formation *in vitro* were partially abolished after *CLEC11A* of the parental hucMSCs was silenced. The results suggest that hucMSC-EVs may enhance bone mass and strength by delivering functional CLEC11A to stimulate osteogenesis and inhibit adipogenesis and osteoclastogenesis. Nevertheless, Yue *et al*. reported that CLEC11A has no effect on bone resorption and adipocyte formation *in vivo* although they do observe significantly larger numbers of adipocyte in 10 month-old *CLEC11A* deficient mice relative to littermate controls 52, which appeared to be inconsistent with our *in vitro* results. Actually, we could not assume whether interference of *CLEC11A* by shRNA in hucMSCs might also have the potential to influence the expression of other genes or alter the molecular components loaded into hucMSC-EVs through some unknown mechanisms to change the regulatory effects of EVs. Therefore, further studies are needed to be conducted to make clear these paradoxical results. Moreover, it is noteworthy that inhibition of CLEC11A did not thoroughly block the beneficial effects of hucMSC-EVs on bone metabolism *in vitro*, indicating the implication of other proteins or RNAs including miRNA, which was consistent with proteomic results that hucMSC-EVs were also abundant in other bone protective proteins. The failure to detect the *in vivo* role of CLEC11A in hucMSC-EVs-induced regulation of bone metabolism may be considered as another limitation of this study.

In conclusion, the current findings demonstrate that hucMSC-EVs can prevent osteoporosis in mice by promoting the differentiation of BMSCs from adipocyte to osteoblast and inhibiting osteoclastogenesis. Furthermore, CLEC11A partially mediates the effect of hucMSC-EVs on bone metabolism. Our results suggest that hucMSC-EVs may represent potential therapeutic agents for prevention and treatment of osteoporosis.

## Supplementary Material

Supplementary figures.

Table S1.

## Abbreviations

BMSCs: bone marrow mesenchymal stromal cells
hucMSCs: human umbilical cord-derived mesenchymal stromal cells
EVs: extracellular vesicles
hucMSC-EVs: EVs derived from hucMSCs
PBS: phosphate buffer saline
FBS: fetal bovine serum
ARS: Alizarin Red S
ORO: Oil Red O
TEM: transmission electron microscope
OVX: ovariectomy
TS: tail suspension
µCT: microcomputed tomography
PFA: paraformaldehyde
ROI: region of interest
H&E: hematoxylin and eosin
OCN: osteocalcin
TRAP: tartrate-resistant acid phosphatase
MAR: mineral apposition rates
ELISA: enzyme-linked immunosorbent assay
CTX-I: C-terminal telopeptides of type I collagen
CLEC11A: C-type lectin domain family 11, member A
ALP: alkaline phosphatase
qRT-PCR: quantitative real-time PCR
Adipo: adiponectin
Retn: resistin
Pparg: peroxisome proliferator-activated receptor-g
RANKL: receptor activator for nuclear factor-κB ligand
Runx2: runt-related transcription factor 2
Sp7: osterix
Col1a1: collagen type I α1
Dmp1: dentin matrix protein 1
Nfatc1: nuclear factor of activated T cells c1
Ctsk: cathepsin K
