# Supplementary material for: Human umbilical cord mesenchymal stromal cells-derived extracellular vesicles exert potent bone protective effects by CLEC11A-mediated regulation of bone metabolism
Source: Theranostics. 2020 Jan 16;10(5):2293–308. doi: 10.7150/thno.39238 (PMC7019162; doi:10.7150/thno.39238)
Supplement: Supplementary file 1 — Supplementary figures. [file thnov10p2293s1.pdf]

Supplementary Material

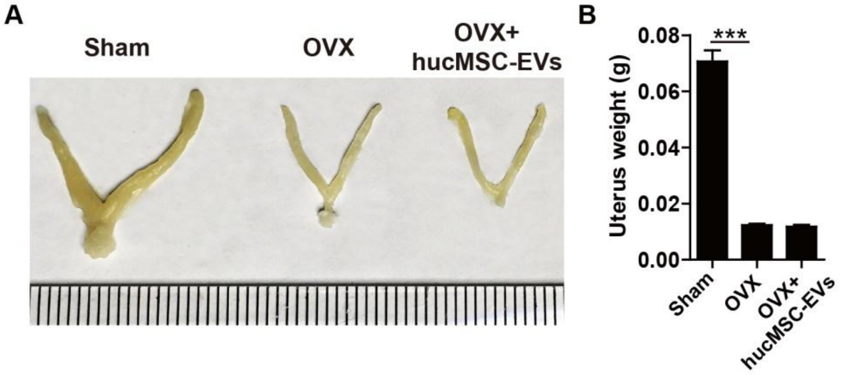

Figure S1. OVX mice show decreased size and weight of uterus. (A) Representative images of uteruses from Sham, OVX and OVX + hucMSC-EVs mice. **(B)** Quantitative analysis of uterus weight in (A). n = 8 per group for OVX + hucMSC-EVs; n = 10 for other groups. *****P < 0.05, ******P < 0.01, *******P < 0.001.

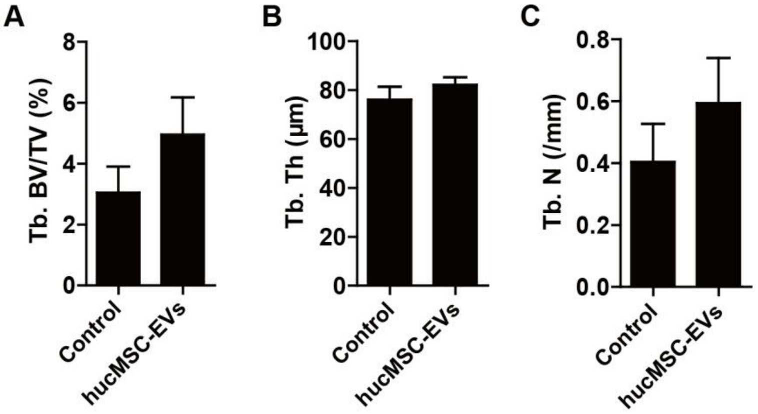

Figure S2. hucMSC-EVs slightly reverse the osteoporotic phenotypes of aged mice. (A-C) Quantitative μCT analysis of Tb. BV/TV, Tb. Th and Tb. N in femora from hucMSC-EVs- or PBS-treated 19-month-old mice. n = 7 per group (PBS); n = 8 per group (hucMSC-EVs).

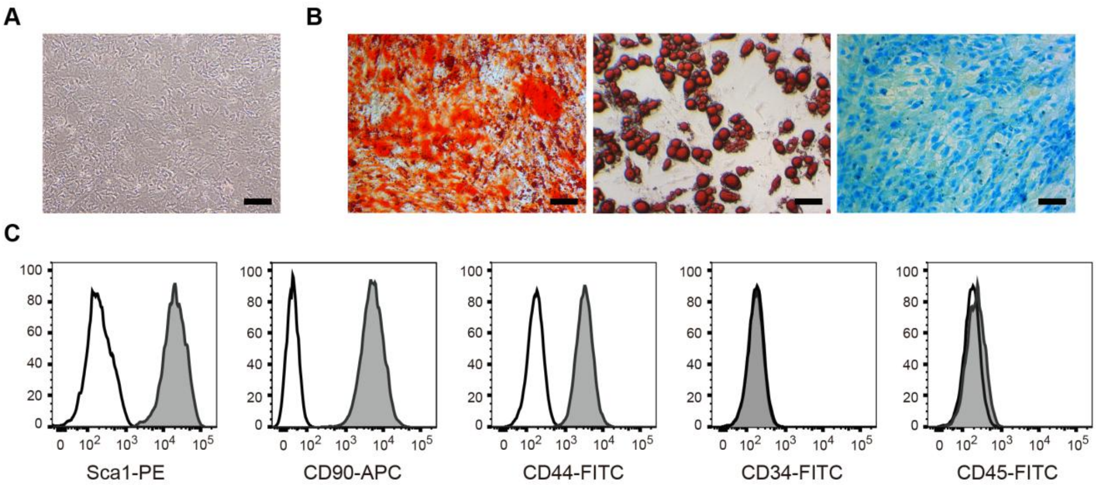

Figure S3. Identification of BMSCs. (A) BMSCs showed a fusiform morphology. Scale bar: 100 μm.

(B) BMSCs had the ability to differentiate into osteoblasts, adipocytes or chondrocytes under osteogenic, adipogenic or chondrogenic culture condition, confirmed by Alizarin Red S staining, Oil Red O staining and Alcian Blue staining. Scale bars: 50 μm. **(C)** The characteristic surface markers on BMSCs analyzed by flow cytometry. Blank curves: the isotype controls; solid gray curves: the test samples.
